# Plasma levels of interleukin-33 and soluble suppression of tumorigenicity 2 in patients with advanced pancreatic ductal adenocarcinoma undergoing systemic chemotherapy

**DOI:** 10.1007/s12032-018-1223-3

**Published:** 2018-11-13

**Authors:** Markus Kieler, Matthias Unseld, Johann Wojta, Alexandra Kaider, Daniela Bianconi, Svitlana Demyanets, Gerald W. Prager

**Affiliations:** 1 Division of Oncology, Department of Medicine I, Comprehensive Cancer Center, Medical University Vienna, Waehringer Guertel 18–20, 1090 Vienna, Austria; 2 Division of Cardiology, Department of Internal Medicine II, Medical University Vienna, Waehringer Guertel 18–20, Vienna, Austria; 3 Center for Medical Statistics, Informatics and Intelligent Systems, Medical University of Vienna, Spitalgasse 23, Vienna, Austria; 4 Department of Laboratory Medicine, Medical University of Vienna, Waehringer Guertel 18–20, 1090 Vienna, Austria

**Keywords:** IL-33, sST2, Cytokines, Pancreatic ductal adenocarcinoma, Chronic inflammation

## Abstract

Interleukin-33 (IL-33) and its “decoy” receptor soluble ST2 (sST2) are involved in the development of chronic inflammation and cancer. We explored IL-33 and sST2 as a potential prognostic marker in patients with metastatic and locally advanced pancreatic ductal adenocarcinoma (PDAC). IL-33 and sST2 plasma levels were assessed in 20 patients with advanced PDAC before start of systemic chemotherapy and were analyzed in relation to clinical outcome. Kaplan Meier and multivariable Cox proportional hazards model analysis revealed a significant association between sST2 plasma levels and survival (HR 2.10, 95% CI 1.33–3.41, *p* = 0.002) and link high sST2 plasma levels to inferior survival in patients with advanced PDAC undergoing chemotherapy.

## Introduction

Pancreatic ductal adenocarcinoma (PDAC) is a lethal disease with a devastating 5-year overall survival of only ~ 7%. Although just 4% of all malignant diseases is accounted to PDAC, it will become the second leading cause of cancer-related deaths before 2030 [1]. A major cause for the aggressiveness and dismal prognosis of this malignant disease is the abundance of immune suppressive mechanisms displayed by PDAC, which is thought to support tumor growth and promote metastasis. The former perception of PDAC as a poorly immunogenic tumor has now been replaced by the notion of an inflamed tumor that evades the immune control by exerting immunosuppressive mechanisms in its microenvironment [2]. For this reason, kinetics of blood-based markers might be used to track the footprint that is left by the inflammatory response in patients with PDAC that reflects the current state of the tumor in terms of tumor growth and progression.

Interleukin-33 (IL-33) is a pro-inflammatory cytokine and it is involved in the development of chronic inflammation and cancer [3–6]. It is negatively regulated by several mechanisms and sequestration by the “decoy” receptor soluble suppression of tumorigenicity 2 (sST2) is likely to be crucial in this process [7]. IL-33 is upregulated in PDAC and nonmalignant cells in the tumor microenvironment of PDAC and lesions from chronic pancreatitis: pancreatic stellate cells and pancreatic myofibroblasts have been identified as important sources of this cytokine [8–10].

Therefore, we assessed the plasma levels of IL-33 and sST2 by blood sampling in patients with PDAC before start of their systemic cytotoxic therapy to determine its role as a prognostic marker in PDAC.

## Methods

### Subjects and study design

This is a single center, retrospective, observational study including patients with histologically proven non-resectable PDAC that was either locally advanced or metastastic. Patients who started first line systemic cytotoxic chemotherapy with albumin-bound paclitaxel (nab-paclitaxel) or later line chemotherapy with nanoliposomal irinotecan (nal-iri) in combination with infusional 5-fluorouracil (5-FU)/leucovorin (LV) in the recruitment period between July 2016 and January 2017 were eligible for this study. The study endpoint was the clinical outcome as determined by efficacy endpoints such as overall survival (OS), objective response rate (ORR), progression free survival (PFS), and disease control rate (DCR). OS was calculated from the time of the start of the therapy until death from any cause. Survival times of patients still alive at the last follow-up visit (in February and March 2018) were considered as censored observations. PFS was calculated from the start of the therapy to disease progression or death from any cause. Again, survival times of patients without disease progression at the last follow-up visit were interpreted as censored observations. ORR was determined by the proportion of patients with an objective response and DCR was determined by the proportion of patients that had an objective response or a stable disease. Computer tomography as efficacy assessment according to RECIST 1.1 was performed at baseline and at week 12. The study was approved by the ethical committee of the Medical University of Vienna (EK 274/2011) and complies with the Declaration of Helsinki. All patients had to provide written informed consent before participating in this study.

### Blood sampling

Blood sampling was performed into EDTA-containing tubes at day one of the first administration of the systemic chemotherapy regimen. Samples were processed within 4 h after collection by centrifugation at 1500 × *g* for 10 min. Plasma samples were then aliquoted and stored at − 80 °C until further use. Carbohydrate Antigen 19-9 (CA 19-9) concentrations was measured using Electro-chemiluminescence immunoassay (ECLIA) on cobas e analyzer (Roche Diagnostics, Mannheim, Germany).

### IL-33 and sST2 measurement

Circulating levels of IL-33 were measured using the Human IL-33 DuoSet® ELISA (R&D Systems, Minneapolis, MN, USA) according to manufacturer’s instructions (sensitivity 1.65 pg/ml), while circulating levels of sST2 were measured using the Human ST2/IL-1 R4 Duoset® ELISA (R&D Systems, Minneapolis, MN, USA) according to manufacturer’s instructions (sensitivity 12.5 pg/ml) as described previously [11–14].

### Statistical analysis

Continuous data are given as median and interquartile range (IQR). Categorical variables are summarized as counts and percentages. The median follow-up time was calculated using the inverse Kaplan–Meier method [15]. Survival curves were estimated by the Kaplan–Meier method and compared using the Log-rank test. Univariate and multivariable Cox proportional hazards regression models were used to evaluate the unadjusted and treatment adjusted effects of IL-33 and sST2 plasma levels on overall survival. Due to their skew distributions, log2-transformed values were considered for statistical analyses. Therefore, estimated hazard ratios (HR) refer to a twofold change in the respective explanatory variable. To further explore the nature of the potential prognostic effect of IL-33 on overall survival, additional univariate and multivariable Cox regression models were performed, considering IL-33 as a binary factor (above versus below the detectable limit of 0.001 pg/ml) alone and combined with an additional continuous factor (including the log2-transformed IL-33 value for patients with IL-33 above the detection limit, and the value zero for patients with IL-33 below the detection limit). To reduce bias in estimates resulting from the small number of patients, the Firth correction was used [16]. The correlation of IL-33, sST2, and CA19-9 values is described by the Pearson correlation coefficient, and the partial Pearson correlation coefficient, respectively. The SAS software version 9.4 (SAS Institute Inc., 2016, Cary, NC, USA) was used for statistical analyses. *P* values < 0.05 were considered as statistically significant.

## Results

### Baseline characteristics of patients

Ten male (50%) and ten female (50%) patients with advanced PDAC participated in this study (Table 1). The median age at time of diagnosis with advanced disease was 62 years (IQR 58–70 years). Fourteen patients (70%) had an Eastern Cooperative Oncology Group (ECOG) performance status of 0 and six patients (30%) had an ECOG performance status of 1. In fourteen patients (70%), the CA19-9 levels were above 40 U/ml and in four patients (20%), the levels were below 40 U/ml. In two patients (10%), baseline CA19-9 levels were not available. Three patients (15%) had locally advanced (stage III) and 17 patients (85%) had metastatic (stage IV) disease. Liver (*n* = 10, 50%) was the predominant metastatic site followed by peritoneum (*n* = 5, 25%) and lung (*n* = 3, 15%). Three patients (15%) presented with additional distant lymph node metastasis. 13 patients (65%) had one and four patients (20%) had two different organs affected from metastatic spread. Half of the patients (*n* = 10, 50%) were therapy naïve and underwent first line systemic chemotherapy with gemcitabine and nab-paclitaxel, whereas the other half of the patients received nal-iri in combination with infusional 5-FU/LV and have previously progressed under a gemcitabine-based chemotherapy. Six patients (30%) had one previous treatment line and four patients (20%) already had two or more lines.

**Table 1:** Baseline characteristics

	*N* = 20 (%)
Sex	
Men	10 (50)
Women	10 (50)
Age at time of diagnosis (years, range)	62 (58–70)
ECOG performance status	
0	14 (70)
1	6 (30)
Amount of CA19-9 at time of first blood sample	
≥ 40 U/ml	14 (70)
< 40 U/ml	4 (20)
n/a	2 (10)
Disease stage	
Metastatic (stage IV)	17 (85)
Locally advanced (stage III)	3 (15)
Site of metastatic lesions	
Liver	10 (50)
Peritoneal	5 (25)
Lung	3 (15)
Other	3 (15)
Number of metastatic sites	
0	3 (15)
1	13 (65)
2	4 (20)
Previous lines of systemic therapy	
0	10 (50)
1	6 (30)
2	3 (15)
> 2	1 (5)
Administered chemotherapy	
nab-paclitaxel/gemcitabine	10 (50)
nal-iri/5-FU/LV	10 (50)

### Clinical outcome

Among ten patients assigned to first line treatment gemcitabine/nab-paclitaxel, two patients (20%) had an objective response, three patients (30%) a stable disease, and five patients (50%) were progressing under systemic chemotherapy. The disease control rate (DCR) and overall response rate (ORR) in these patients were 50% and 20%. Median progression free survival (mPFS) was 6.5 months (95% CI 1.2—not estimable) and median overall survival (mOS) was 15.8 months (95% CI 1.2—not estimable). From the ten patients undergoing treatment with nal-iri/5-FU/LV, one patient (10%) had an objective response, four patients (40%) had a stable disease, and five patients (50%) were progressing. The DCR and ORR in these patients were 50% and 10%. MPFS and mOS was 3.1 months (95% CI 2.1–6.0) and 6.3 months (95% CI 2.8–10.2), respectively. Results are shown in Table 2.

**Table 2:** Median overall survival, median progression-free survival, and response rates

	Patients treated with gemcitabine/nab-paclitaxel (*n* = 10)	Patients treated with nal-iri/5-FU/LV (*n* = 10)	All patients (*n* = 20)
mPFS in months (95% CI)	6.5 (1.2–n.e.)	3.1 (2.1–6.7)	6.1 (2.8–7.4)
mOS in months (95% CI)	15.8 (1.2–n.e.)	6.4 (2.8–10.2)	7.8 (4.1–15.8)
ORR (%)	20	10	30
DCR (%)	50	50	50

*mPFS* median progression-free survival, *mOS* median overall survival, *ORR* overall response rate, *DCR* disease control rate, *n.e*. not estimable, *CI* confidence interval

### Levels of IL-33 or sST2 and survival

Median plasma levels of IL-33 and sST2 for the entire cohort of patients were 6.8 pg/ml (IQR 0.0–33.6) and 13,064 pg/ml (IQR 6916–22,262), respectively. Median follow-up time was 18.2 months. Kaplan Meier analysis and Cox proportional hazards model revealed a significant association of IL-33 and sST2 plasma levels with OS.

**Fig. 1:**
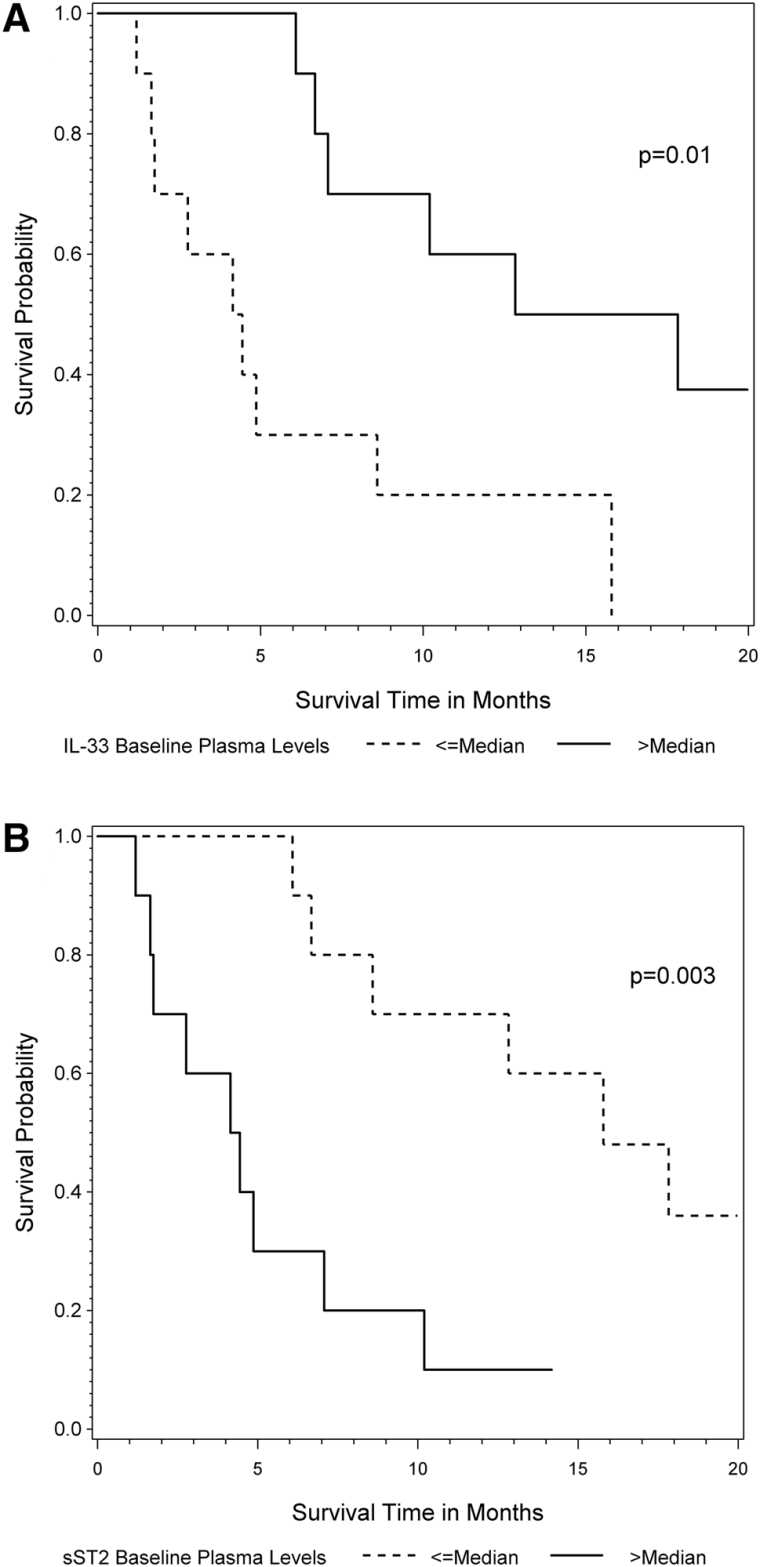
Survival according to plasma levels of IL-33 and sST2. Kaplan Meier survival curves in patients with IL-33 (**a**) and sST2 (**b**) plasma levels above (solid line) and below or equal to the median (dotted line), respectively. Sample size for curves are 20 patients. Median OS for IL-33 plasma levels was 4.3 versus 15.3 months (*p* = 0.01) and for sST2 plasma levels 15.8 versus 4.3 months (*p* = 0.003)

Patients with plasma levels of IL-33 that were less than or equal to the median had a mOS of 4.3 months (95% CI 1.2–8.6), whereas patients with IL-33 plasma levels higher than the median had a mOS of 15.3 months (95% CI 6.1—not estimable, *p* = 0.01) (Fig. 1a). To adjust for a possible effect of first line versus later line chemotherapy, a Cox proportional hazards model was used. High IL-33 plasma levels were significantly associated with a superior survival in the univariate (HR 0.86, 95% CI 0.76–0.96, *p* = 0.008) and multivariable treatment adjusted model (HR 0.82, 95% CI 0.71–0.93, *p* = 0.002). Due to IL-33 plasma levels lower than the detectable limit in seven patients, we further aimed to analyze whether the survival differed in patients with IL-33 plasma levels lower versus above the detectable limit. Indeed, the binary factor (IL-33 plasma levels ≥ 0.001 vs. < 0.001 pg/ml) showed a statistical significant effect on the survival in the univariate (HR 0.19, 95% CI 0.07–0.57; *p* = 0.004) as well as the multivariable treatment adjusted model (HR 0.14, 95% CI 0.04–0.46; *p* = 0.001). However, there was no statistically significant difference in survival in patients with IL-33 plasma levels over the detectable limit in relation to the continuous levels (HR 0.93, 95% CI 0.76–1.18, *p* = 0.54).

Regarding sST2-plasma levels and survival, there was a statistical significant difference in survival for patients with plasma levels below or equal to the median with a mOS of 15.8 months (95% CI 6.1—not estimable) versus over the median with a mOS of 4.3 months (95% CI 1.2–7.1, *p* = 0.003) (Fig. 1b). This survival difference was confirmed in the univariate (HR 2.08, 95% CI 1.36–3.30, *p* = 0.001) and multivariable (HR 2.10, 95% CI 1.33–3.41, *p* = 0.002) treatment adjusted Cox proportional hazards model.

### Correlation of IL-33, sST2, and CA19-9

To investigate a possible correlation of IL-33, sST2 plasma levels and the clinically well-established serum tumor marker CA19-9, the correlation coefficient was calculated by using the Pearson correlation. Plasma levels for IL-33, sST2, and serum levels for CA19-9 were correlated to each other. There was a moderate negative correlation of IL-33 and sST2 plasma levels (rho = − 0.449, *p* = 0.047). Neither plasma levels of IL-33 and sST2 correlated with serum levels of CA19-9. Results are shown in Table 3.

**Table 3:** Correlation of plasma or serum levels of IL-33, sST2, and CA19-9

	IL-33	sST2	CA19-9
Correlation of plasma or serum levels of IL-33, sST2 and CA19-9			
IL-33		− 0.449 (0.047)	− 0.238 (0.342)
sST2			+ 0.263 (0.292)

The table shows the results for the Pearson correlation coefficient, which was calculated using the Log2 values of the plasma or serum levels

*p* values are shown in parentheses

## Discussion

This is the first study that evaluated circulating IL-33 and sST2 as a potential prognostic marker in patients with advanced PDAC.

There is increasing evidence that the IL-33/sST2 axis plays a crucial role in tumorigenesis and tumor progression in various malignancies such as colon, head and neck, breast, gastric, ovarian, lung, renal and pancreas [10, 17–25]. IL-33 has diverse context-dependent functions but originally it has been found that it mediates its biological effects via IL-1 family receptor transmembrane ST2 (ST2L) and activates NF-kB and MAP kinases. Its administration leads to significant increases in gene expression of prominent T helper (Th)_2_-associated cytokines IL-4, IL-5, and IL-13 in vivo in mice [26]. In PDAC, there is a strong imbalance between Th_1_ and Th_2_ response toward a Th_2_-type response and tumor infiltration with Th_2_-lymphocytes correlates with an increased expression pattern of Th_2_-related cytokines in the blood of PDAC patients [27, 28]. A predominant Th_2_-infiltrate in the tumor is an independent predictive marker of poor survival in PDAC patients [29]. To date, all studies except of one have linked increased serum levels of IL-33 and sST2 in cancer patients to negative prognosis [30–36]. For example, decreased survival has been demonstrated in non-small cell lung cancer patients with high serum levels of IL-33 and in hepatocellular carcinoma patients with high serum levels of sST2 [37, 38]. Our results support that high plasma levels of sST2 are also associated with inferior survival of PDAC patients undergoing systemic chemotherapy.

Regarding our results for IL-33, we found a significant negative effect of low IL-33 plasma levels on survival; however, in 35% of all patients, the measured IL-33 levels where below the detectable limit of the assay. Therefore, more caution to the interpretation of linking IL-33 plasma levels to survival has to be paid. If the patients are split up into two groups (IL-33 plasma levels < 0.001 vs. ≥ 0.001 pg/ml), we observed a statistically significant negative effect on survival in the univariate as well as in the multivariable treatment adjusted model. However, in the same model, there was no statistically significant difference in survival in patients with IL-33 plasma levels over the detectable limit in relation to the continuous levels (HR 0.93, 95% CI 0.76–1.18, *p* = 0.54) neither in the univariate nor in the multivariable model. Therefore, we found no statistical evidence suggesting that IL-33 plasma levels over the detectable limit have any effect on the survival in our patients. It must also be mentioned, that currently available IL-33 ELISA assays still lack the necessary reliability that would be needed for valid survival analysis [39]. In connection to that, we also just found a moderate negative correlation of IL-33 and sST2 levels. Recent studies have described pancreatic stellate cells and pancreatic myofibroblasts as sources of IL-33 production [8, 9]. As an intracellular cytokine, IL-33 is released after cell or tissue damage [40]. When released, IL-33 could enhance the expression of chemokines and cytokines in pancreatic myofibroblasts and stimulate the proliferation and migration of these cells, which further contributes to the pathogenesis of pancreatic desmoplasia, a hallmark of PDAC [8, 41, 42]. However, some studies also propose a possible protective role of the IL33/sST2 axis in melanoma, breast, colorectal, prostate and hepatocellular cancer tumor models by stimulating antitumor immunity [43–48]. Therefore, further work is required to clarify the role of IL-33 in cancer.

The greatest limitations of our study are the small sample size, its retrospective character, and the current lack of a reliable IL-33 ELISA assay. If IL-33 is to be studied in the serum of patients, a more sensitive and specific assay method will be required, which is vital for further understanding and targeting of the IL-33/IL-1RL1 axis in human disease. This issue has been addressed in a recent study comparing currently available IL-33 ELISA assays [39]. With a larger patient cohort and a more reliable IL-33 assay, we would also not only expect to clarify the prognostic relevance of IL-33 plasma levels in PDAC patients but we could also divide patients into four different groups according to IL-33 and sST2 levels (IL-33^low^/sST2^low^, IL-33^high^/sST2^low^, IL-33^low^/sST2^high^, IL-33^high^/sST2^high^) to further elucidate the role of the relation between IL-33 and sST2 in malignant disease. However, it has to be highlighted that our data demonstrates a clear clue for the prognostic potential of sST2 levels for mortality prediction in our cohort and the statistical significance of the observed effect is also maintained in the treatment adjusted multivariable model.

In conclusion, we here report the first study that measured circulating IL-33 and sST2 in advanced PDAC patients that underwent systemic chemotherapy. Our results suggest a negative impact of high sST2 plasma levels on survival.

## Abbreviations

5-FU: 5-fluorouracil
DCR: Disease control rate
ECOG: Eastern Cooperative Oncology Group
HR: Hazard ratio
IL: Interleukin
IQR: Interquartile range
LV: Leucovorin
Nab-paclitaxel: Albumin-bound paclitaxel
Nal-iri: Nanoliposomal irinotecan
ORR: Overall response rate
OS: Overall survival
PDAC: Pancreatic ductal adenocarcinoma
PFS: Progression free survival
sST2: Soluble suppression of tumorigenicity 2
Th: T helper

## References

[1] Rahib L, Smith BD, Aizenberg R (2014). Projecting cancer incidence and deaths to 2030: the unexpected burden of thyroid, liver, and pancreas cancers in the united states. Cancer Res.

[2] Zheng L, Xue J, Jaffee EM (2013). Role of immune cells and immune-based therapies in pancreatitis and pancreatic ductal adenocarcinoma. Gastroenterology.

[3] Liew FY, Girard J, Turnquist HR (2016). Interleukin-33 in health and disease. Nat Rev Immunol.

[4] Demyanets S, Konya V, Kastl SP (2011). Interleukin-33 induces expression of adhesion molecules and inflammatory activation in human endothelial cells and in human atherosclerotic plaques. Arterioscler Thromb Vasc Biol.

[5] Stojkovic S, Kaun C, Heinz M (2014). Interleukin-33 induces urokinase in human endothelial cells–possible impact on angiogenesis. J Thromb Haemost.

[6] Montanari E, Stojkovic S, Kaun C (2016). Interleukin-33 stimulates GM-CSF and M-CSF production by human endothelial cells. Thromb Haemost.

[7] Sanada S, Hakuno D, Higgins LJ (2007). IL-33 and ST2 comprise a critical biomechanically induced and cardioprotective signaling system. J Clin Invest.

[8] Nishida A, Andoh A, Imaeda H (2010). Expression of interleukin 1-like cytokine interleukin 33 and its receptor complex (ST2L and IL1RAcP) in human pancreatic myofibroblasts. Gut.

[9] Masamune A, Watanabe T, Kikuta K (2010). Nuclear expression of interleukin-33 in pancreatic stellate cells. Am J Physiol Gastrointest Liver Physiol.

[10] Mohammed A, Janakiram NB, Madka V (2017). Lack of chemopreventive effects of P2 × 7R inhibitors against pancreatic cancer. Oncotarget.

[11] Demyanets S, Speidl WS, Tentzeris I (2014). Soluble ST2 and Interleukin-33 levels in coronary artery disease: relation to disease activity and adverse outcome. PLoS ONE.

[12] Demyanets S, Tentzeris I, Jarai R (2014). An increase of interleukin-33 serum levels after coronary stent implantation is associated with coronary in-stent restenosis. Cytokine.

[13] Krychtiuk KA, Stojkovic S, Lenz M (2018). Predictive value of low interleukin-33 in critically ill patients. Cytokine.

[14] Stojkovic S, Kaider A, Koller L (2018). GDF-15 is a better complimentary marker for risk stratification of arrhythmic death in non-ischaemic, dilated cardiomyopathy than soluble ST2. J Cell Mol Med.

[15] Schemper M, Smith TL (1996). A note on quantifying follow-up in studies of failure time. Control Clin Trials.

[16] Heinze G, Schemper M (2001). A solution to the problem of monotone likelihood in Cox regression. Biometrics.

[17] Chen SF, Nieh S, Jao SW (2013). The paracrine effect of cancer-associated fibroblast-induced interleukin-33 regulates the invasiveness of head and neck squamous cell carcinoma. J Pathol.

[18] Liu X, Zhu L, Lu X (2014). IL-33/ST2 pathway contributes to metastasis of human colorectal cancer. Biochem Biophys Res Commun.

[19] Cui G, Qi H, Gundersen MD (2015). Dynamics of the IL-33/ST2 network in the progression of human colorectal adenoma to sporadic colorectal cancer. Cancer Immunol Immunother.

[20] Kim JY, Lim S-C, Kim G (2015). Interleukin-33/ST2 axis promotes epithelial cell transformation and breast tumorigenesis via upregulation of COT activity. Oncogene.

[21] Yu XX, Hu Z, Shen X (2015). IL-33 promotes gastric cancer cell invasion and migration via ST2-ERK1/2 pathway. Dig Dis Sci.

[22] Tong X, Barbour M, Hou K (2016). Interleukin-33 predicts poor prognosis and promotes ovarian cancer cell growth and metastasis through regulating ERK and JNK signaling pathways. Mol Oncol.

[23] Akimoto M, Hayashi J-I, Nakae S (2016). Interleukin-33 enhances programmed oncosis of ST2L-positive low-metastatic cells in the tumour microenvironment of lung cancer. Cell Death Dis.

[24] Wang Z, Xu L, Chang Y (2016). IL-33 is associated with unfavorable postoperative survival of patients with clear-cell renal cell carcinoma. Tumor Biol.

[25] Schmieder A, Multhoff G, Radons J (2012). Interleukin-33 acts as a pro-inflammatory cytokine and modulates its receptor gene expression in highly metastatic human pancreatic carcinoma cells. Cytokine.

[26] Schmitz J, Owyang A, Oldham E (2005). IL-33, an interleukin-1-like cytokine that signals via the IL-1 receptor-related protein ST2 and induces T helper type 2-associated cytokines. Immunity.

[27] Bellone G, Turletti A, Artusio E (1999). Tumor-associated transforming growth factor-beta and interleukin-10 contribute to a systemic Th2 immune phenotype in pancreatic carcinoma patients. Am J Pathol.

[28] Tassi E, Gavazzi F, Albarello L (2008). Carcinoembryonic antigen-specific but not antiviral CD4 + T cell immunity is impaired in pancreatic carcinoma patients. J Immunol.

[29] De Monte L, Reni M, Tassi E (2011). Intratumor T helper type 2 cell infiltrate correlates with cancer-associated fibroblast thymic stromal lymphopoietin production and reduced survival in pancreatic cancer. J Exp Med.

[30] Kim MS, Kim E, Heo JS (2015). Circulating IL-33 level is associated with the progression of lung cancer. Lung Cancer.

[31] Yang ZP, Ling DY, Xie YH (2015). The association of serum IL-33 and sST2 with breast cancer. Dis Markers.

[32] Bergis D, Kassis V, Radeke HH (2016). High plasma sST2 levels in gastric cancer and their association with metastatic disease. Cancer Biomarkers.

[33] Sun P, Ben Q, Tu S (2011). Serum Interleukin-33 levels in patients with gastric cancer. Dig Dis Sci.

[34] Lu D, Zhou X, Yao L (2014). Serum soluble ST2 is associated with ER-positive breast cancer. BMC Cancer.

[35] Zeng X, Zhang Z, Gao Q-Q (2016). Clinical significance of serum interleukin-31 and interleukin-33 levels in patients of endometrial cancer: a case control study. Dis Markers.

[36] Zhang P, Liu X-K, Chu Z (2012). Detection of interleukin-33 in serum and carcinoma tissue from patients with hepatocellular carcinoma and its clinical implications. J Int Med Res.

[37] Hu L-A, Fu Y, Zhang D-N (2013). Serum IL-33 as a diagnostic and prognostic marker in non- small cell lung cancer. Asian Pac J Cancer Prev.

[38] Bergis D, Kassis V, Ranglack A (2013). High serum levels of the interleukin-33 receptor soluble ST2 as a negative prognostic factor in hepatocellular carcinoma. Transl Oncol.

[39] Ketelaar ME, Nawijn MC, Shaw DE (2016). The challenge of measuring IL-33 in serum using commercial ELISA: lessons from asthma. Clin Exp Allergy.

[40] Cayrol C, Girard JP (2014). IL-33: an alarmin cytokine with crucial roles in innate immunity, inflammation and allergy. Curr Opin Immunol.

[41] Theocharis AD, Tsara ME, Papageorgacopoulou N (2000). Pancreatic carcinoma is characterized by elevated content of hyaluronan and chondroitin sulfate with altered disaccharide composition. Biochim Biophys Acta.

[42] Kadaba R, Birke H, Wang J (2013). Imbalance of desmoplastic stromal cell numbers drives aggressive cancer processes. J Pathol.

[43] Gao X, Wang X, Yang Q (2015). Tumoral expression of IL-33 inhibits tumor growth and modifies the tumor microenvironment through CD8 ^+^ T and NK cells. J Immunol.

[44] Akimoto M, Maruyama R, Takamaru H (2016). Soluble IL-33 receptor sST2 inhibits colorectal cancer malignant growth by modifying the tumour microenvironment. Nat Commun.

[45] Saranchova I, Han J, Huang H (2016). Discovery of a metastatic immune escape mechanism initiated by the loss of expression of the tumour biomarker interleukin-33. Sci Rep.

[46] Brunner SM, Rubner C, Kesselring R (2015). Tumor-infiltrating, interleukin-33-producing effector-memory CD8^+^ T cells in resected hepatocellular carcinoma prolong patient survival. Hepatology.

[47] Fang Y, Zhao L, Xiao H (2017). IL-33 acts as a foe to MIA PaCa-2 pancreatic cancer. Med Oncol.

[48] Serrels B, McGivern N, Canel M (2017). IL-33 and ST2 mediate FAK-dependent antitumor immune evasion through transcriptional networks. Sci Signal.

